# Study on the Distribution Characteristics and Risk Assessment of Antibiotics and Resistance Genes in Water Sources of Wuhan

**DOI:** 10.3390/toxics12070507

**Published:** 2024-07-14

**Authors:** Jun Wang, Ying Yu, Jiayi Jiang, Bolin Li, Weimin Xie, Gezi Li, Huanjie Song, Wanying Zhai, Ye Li

**Affiliations:** 1 School of Resource and Environmental Engineering, Wuhan University of Technology, Wuhan 430070, China; wjunwuhan@163.com (J.W.); 17612748023@163.com (Y.Y.); 15612143556@163.com (G.L.); whly1218@126.com (Y.L.); 2 Wuhan Lingang Economic and Technological Development Zone Service Industry Development Investment Group Co., Ltd., Wuhan 430040, China; m18336851069@163.com; 3 Powerchina Eco-Environmental Group Co., Ltd., Shenzhen 518102, China; jiangjy-shj@powerchina.cn; 4 Changjiang Basin Ecology and Environment Monitoring and Scientific Research Center, Changjiang Basin Ecology and Environment Administration, Ministry of Ecology and Environment, Wuhan 430010, China; wyzhai@iccas.ac.cn

**Keywords:** antibiotic, ARGs, drinking water, spatiotemporal distribution, risk assessment

## Abstract

In contemporary society, the improper use of antibiotics leads to their persistent presence in the ecological environment. Due to the diverse physical and chemical properties of antibiotics, their spatial and temporal distribution in the environment varies. Moreover, antibiotics can stimulate the emergence of antibiotic resistance genes (ARGs), which complicates the monitoring and regulation of antibiotics and poses a significant threat to both aquatic and terrestrial environments. This study investigated the distribution of 15 antibiotics and 11 typical ARGs across four categories at 19 sites of drinking water sources in Wuhan, China. The findings revealed that the concentration of antibiotics during the dry season (nd~61,883 ng/L) was significantly higher compared to both the normal water season (nd~49,883 ng/L) and the wet season (nd~28,686 ng/L). Sulfamethoxazole (SMX), sulfamethoxazole (SMD), sulfadiazine (SD), and roxithromycin (RTM) were the predominant antibiotics in the target water environments. The study indicated that most of the antibiotics analyzed posed little to no risk to aquatic organisms. The primary ARGs detected in the surface water of the study area were *sul1*, *qnrD*, and *tetO*. Furthermore, some ARGs showed a negative correlation with their respective antibiotics. Additional research is necessary to evaluate the impact of these emerging pollutants (antibiotics and ARGs) on the safety of high-quality drinking water for residents in Wuhan City.

## 1. Introduction

Since the discovery of penicillin by Fleming in the last century, antibiotics have increasingly been extensively utilized in human activities and daily life. These powerful drugs have played a pivotal role in the prevention and treatment of diseases on a global scale, effectively combating zoonotic diseases, and significantly advancing the rapid development of agriculture, animal husbandry, and aquaculture [1,2,3]. Due to incomplete digestion and absorption of antibiotics in humans and animals, the majority of these medications are excreted from the body in the form of unchanged parent compounds or metabolites through urine and feces. Additionally, they can enter the sewage treatment system through various channels, including rain washing and surface runoff [4,5,6,7]. With the substantial use of antibiotics, antibiotic pollution has become a global problem. Antibiotic residues are frequently detected in many domestic and international rivers [8,9,10,11], lakes [12,13,14], reservoirs [11,15], and oceans [16,17]. The removal rate of antibiotic pollutants using traditional sewage treatment processes is lower than 80% [18], and the residual antibiotics are discharged into the water environment with the effluent. Furthermore, antibiotics can be accumulated in organisms [19,20,21], thereby indirectly entering the human body and posing a threat to the ecological environment and humans.

Moreover, the persistent existence of antibiotics results in the emergence and spread of ARGs. This phenomenon occurs even at low concentrations of antibiotics (ng/L~μg/L) in aquatic environments. Even trace amounts of antibiotics can facilitate the horizontal gene transfer (HGT) of ARGs [22,23], while HGT can result in the enhanced spread and contamination of antibiotic resistance genes (ARGs), so it is important to recognize that ARGs persist, migrate, undergo transformation, and disseminate within the natural environment. The potential environmental damage caused by ARGs exceeds that of antibiotics, as they directly impact the natural environment and human ecology. Additionally, they possess the ability to generate “super bacteria”, thereby posing greater health risks and exacerbating the overall threat to the ecosystem [24,25,26].

Currently, the majority of research on antibiotics and ARGs focuses on rivers and lakes. However, there is limited research on the distribution and transformation of antibiotics and ARGs in urban drinking water systems, particularly in water intake systems. Therefore, it is crucial to study the pollution characteristics, migration, and transformation patterns of antibiotics and ARGs in the drinking water system. This research is necessary to develop advanced treatment technologies that specifically target and effectively control antibiotic and ARG pollution.

Studies have shown that the antibiotic pollution level of drinking water sources in the upper Nanjing section of the Yangtze River is ND~dozens of ng/L [27,28]. Current literature has reported on the residues of antibiotics and ARGs in the Yangtze and Han River [10,29,30], most antibiotics and ARGs are primarily found in the surface water and sediments of the Yangtze River within reach of Wuhan. However, there is limited information available regarding the concentration, distribution, potential sources, and water-related risks associated with different types of antibiotics and ARGs in this area. This presents an opportunity to assess the ecological risks posed by antibiotics to various aquatic organisms and the development of antibiotic resistance. Such information can be used to effectively prevent and control antibiotic contamination at a regional level. Additionally, previous studies have often overlooked high-risk antibiotics, which should be given priority in risk management.

Wuhan is abundant in water resources. The Yangtze River and Han River, which constitute the fundamental framework of Wuhan’s extensive water system, also serve as essential water sources for the city. The Han River, as the principal tributary of the Yangtze River, plays a crucial role in providing water to the residents living along its banks, meeting their daily life requirements, and supporting industrial and agricultural endeavors. In this study, fifteen examples of four types of antibiotics, namely sulfonamides (SAs), tetracyclines (TCs), macrolides (MLs), and quinolones (FQs), were quantitatively analyzed using solid-phase extraction–ultra performance liquid chromatography–tandem mass spectrometry (SPE–UPLC–MS/MS), and the corresponding eleven ARGs were quantified using the quantitative PCR method. The primary objective of this study was to examine the temporal and spatial distribution of antibiotics and (ARGs) in 19 drinking water sources in Wuhan during the wet, dry, and normal water seasons. The study seeks to investigate the correlation between these two pollutants and evaluate the potential risks associated with antibiotics.

## 2. Materials and Methods

### 2.1. Chemical Substances and Standards

In this study, four types of antibiotics were selected, including six SAs, four TCs, three FQs, and two MLs, for a total of 15 target antibiotics. Four isotope-labeled antibiotics were spiked into the samples, and atrazine-^13^C_3_ was used as the internal standard to facilitate the quantification method. The study selected 11 target ARGs and used 16S rDNA as the bacterial reference gene. Due to the potential impact of type I integrons on the contagion of ARGs, the test for the integron *intI1* was integrated into the experiment. Details of the antibiotics and resistance genes are provided in Supplementary Text S1. Table 1 shows details of the target antibiotics. Table 2 shows details of the target ARGs.

### 2.2. Sampling Points and Sample Collection

**Figure 1:**
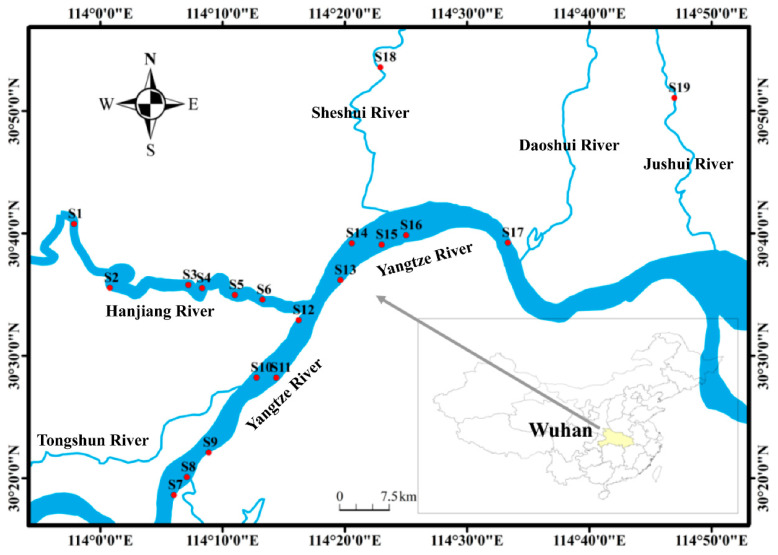
Geographical locations of the sampling sites and sections.

Taking the drinking water sources in Wuhan as the research object, 19 sampling points were selected. The specific locations are shown in Figure 1. Among them, S1~S6 are located in the Hanjiang River, S7~S17 are located in the Yangtze River, and S18~S19 are located in SheShui and JuShui. Supplementary Text S2 shows details of the sampling points. Supplementary Table S1 shows the longitude and latitude.

Three sampling activities were carried out in August 2020, December 2020, and March 2021, encompassing the wet, dry, and normal water seasons of the study area, respectively. Water samples were collected in 3 brown glass bottles with a capacity of 1 L at each point. In order to ensure accurate analysis of tetracycline and sulfonamide antibiotics in water, disodium edetate (0.25 g) and ascorbic acid (0.15 g) were added to the sampling bottle prior to sample collection. This addition effectively eliminated potential interferences from metal ions, residual chlorine, and oxidizing organic compounds.

### 2.3. Antibiotic Quantification

The antibiotic concentrations of the aqueous samples were determined according to previously published methods [31]. Enrichment of antibiotics in water samples through solid-phase extraction: the water samples underwent filtration 0.45 μm by Oasis HLB cartridge (Waters, Milford, MA, USA) on an automated solid-phase extraction device. Detection of antibiotic concentrations: the antibiotic solution obtained from the pretreatment was analyzed by ultra-high performance liquid chromatography–tandem mass spectrometer, and the antibiotic concentration was quantitatively determined using the internal standard method. The specific operation procedure, sample solid phase extraction parameters, instrument gradient elution procedure, mass spectrum information, regression equation, and linear range are shown in Supplementary Text S3. The specific parameters are shown in Supplementary Tables S2~S5.

### 2.4. DNA Extraction and ARG Quantification

According to the method previously published by Jiang et al. [32], vacuum filtration was used to enrich microorganisms in water samples, and then the filter membrane was collected for DNA extraction and analysis. The DNA content and purity were measured by a trace nucleic acid protein analyzer Nanodrop 2000 (Thermo Fisher Scientificanufacture, Waltham, MA, USA), and the concentration of ARGs was detected by qPCR. Sample-specific extraction procedures and instrument analysis parameters are detailed in Supplementary Text S4, and details of primers can be found in Supplementary Table S6.

### 2.5. Ecological Risk Assessment

To quantify the toxicity of residual antibiotics in the aquatic environment on organisms in the aquatic ecosystem, fish, water fleas, and green algae were chosen as representatives of vertebrates, invertebrates, and photosynthetic organisms, respectively. Three different nutritional levels were employed, and the risk entropy value was utilized. The risk quotients (RQs) model was employed to evaluate the potential risks of pollutant residues in water bodies, which were calculated using Equations (1) and (2).

RQ = MEC/PNEC (1)

PNEC = LC50 or EC50 or ChV/AF (2)

In this model, the measured concentration of a drug in the environment (MEC) is compared to the predicted ineffective concentration in water (PNEC). PNEC is defined as the highest drug concentration (ng/L) that is not expected to cause harm to organisms or ecosystems in the environment based on current knowledge; LC50 and EC50 are polar toxic concentrations, which represent the half-lethal concentration and half-inhibitory concentration, respectively; and ChV is the chronic toxic concentration. The minimum result from the three organisms of the same type was used for calculation. AF is a dimensionless evaluation factor referring to the recommended EU “Water Framework Directive” for acute toxicity and chronic toxicity values of 1000 and 100, respectively. According to the RQ value, the ecological risk level is divided into four levels: no risk (RQ ≤ 0.01), low risk (0.01 ≤ RQ < 0.10), medium risk (0.1 ≤ RQ < 1.00), and high risk (RQ > 1.00).

### 2.6. Statistical Analysis

ArcGIS 10.8.2 (ArcMAP) (ESRI, Redlands, CA, USA) was utilized to create the map indicating the locations of the sampling sites. Origin 2021 software (OriginLab, Northampton, MA, USA) was used to illustrate the distribution of antibiotics and resistance genes, and SPSS 22.0 software (SPSS; Chicago, IL, USA) was used to analyze the correlation between antibiotics and ARGs. The correlation between the two variables was assessed using the Pearson correlation coefficient, and a heat map was generated to determine the presence of a relationship between them. Specifically, significance levels of *p* < 0.05 and *p* < 0.01 were considered to indicate significant and highly significant differences between the groups, respectively. The risk entropy value (RQs) model was used to evaluate the potential risks associated with antibiotic contaminants in the water body, and the ECOSAR simulation method was used to calculate the toxicity value of the target antibiotic.

## 3. Results and Discussion 

### 3.1. Spatiotemporal Distribution of Antibiotics

A total of 10 antibiotics were detected in the wet (August), dry (December), and normal water (March of the following year) seasons in the surface water of Wuhan’s water sources, and all four types of antibiotics were detected. The concentration ranges of antibiotics in the three seasons were found to be ND~28.686 ng/L, ND~61.883 ng/L, and ND~49.883 ng/L, respectively. SMX, SMD, RTM, and SD were identified as the main antibiotics in the target water bodies.

The antibiotics that were detected at all nineteen sample points over the three samples periods are shown in Supplementary Figure S1 and summarized in Table 3. Information on temperature, pH, total nitrogen, total phosphorus, and chemical oxygen demand (COD_Mn_) at each sampling site is shown in Supplementary Table S1 and Figure S2.

It is evident from Table 1 that a total of ten antibiotics were detected in the water samples during the wet season, consisting of three SAs, three TCs, three FQs, and one ML. Compared to the wet season, fewer types of target antibiotics were detected during the dry season, and neither TC nor CTC found. This variation could be attributed to the rapid water flow during the wet season, which might dilute the antibiotic residues and allow for the detection of a broader variety of antibiotics. In contrast, during the dry season, the slower water flow may have led to higher concentrations of antibiotic residues, thus reducing the variety of antibiotics detected [33]. In addition, the seasonal variations in dryness and humidity in the Yangtze River basin may also have impacted the biome structure and environmental conditions in the water body, potentially leading to the presence of different species of bacteria and other microorganisms in the water body during different seasons. Consequently, antibiotic testing conducted in different seasons may identify different antibiotic types. In general, the detection of antibiotics in drinking water sources in Wuhan is comparable to that in surface water of the Nanjing section of the Yangtze River and is similar to the concentration results in the water at the mouth of the Yangtze River. The concentrations detected were slightly lower than those found in other parts of river, such as the Nanjing section of the Yangtze River [34,35,36].

In terms of the overall concentration, the antibiotic levels detected in the water source area followed a pattern: dry season > normal water season > wet season. On the one hand, the variability in antibiotic concentration distributions is linked to precipitation [37]. The water flow increases during the wet season, leading to lower pollutant concentrations due to dilution. Conversely, in December, when the water flow is minimal and the flow rate is slow, the antibiotic concentrations are high. Additionally, these variations are also influenced by temperature and light intensity. For instance, during the wet season in August, the higher temperatures and stronger light intensity enhance microbial activity, thereby facilitating both biological degradation and non-biological degradation processes [11], which may result in decreased antibiotic concentrations during the wet season. Additionally, antibiotic concentrations might also be related to seasonal drug consumption, as studies have shown that humans and animals are more susceptible to bacterial infections during winter [38]. Consequently, total antibiotic concentrations peak in December.

In terms of antibiotic types, the three periods overall presented Sas > FQs > TCs > MLs. The dominant antibiotic type in this area was SAs, and the dominant antibiotics were sulfonamides. The findings from studies conducted by Hu et al. [10] on the Han River and Wu et al. [34] on the Yangtze River align with the results of this study. This prevalence can be attributed to the primary use of SA antibiotics in medical applications and their extensive use as antibacterial agents in poultry and aquaculture. These antibiotics are hydrophilic and exhibit high stability, which allows them to readily enter the water environment through runoff and rain erosion [10,39]. In contrast to SAs, the remaining three types of antibiotics have lower detection concentration and frequency, which may be related to their characteristics. The removal rate of FQ antibiotics in sewage plants is high, their persistence in water is relatively low, and they strongly adsorb to solid phases (sediments, suspended particles); TC antibiotics have the characteristics of strong binding ability and cation interaction with particles; and MLs have high hydrophobicity and strong adsorption to soil and sediments [11]. This may provide an explanation for the low concentrations of the three types of antibiotics found in the surface water.

The frequency of detecting RTM did not vary significantly across the three periods, with the highest concentration observed during the normal water season. It is hypothesized that this may be attributed to the high prevalence of influenza in spring and the frequent occurrence of respiratory diseases in humans. Consequently, RTM is likely to be extensively used for treating respiratory tract infections, resulting in the highest concentration during the normal water season [40]. However, due to the COVID-19 pandemic in December 2020 and March 2021, the use of quinolone antibiotics was restricted to cases with highly suspected or confirmed viral infections, leading to a higher frequency of FQ detection compared to the wet season [41]. The average concentration of TC was detected during the wet season, which can be attributed to the high levels of precipitation in the summer, increased temperature, temperature fluctuations, and an increased likelihood of livestock disease. Tetracycline is commonly used as a growth promoter in animal husbandry and aquaculture, as well as being added to animal feed. These practices aim to enhance animal development and address animal illnesses [42].

In terms of the overall spatial distribution, the average concentration of antibiotics in the tests was as follows: JuShui > SheShui > Han River > Yangtze River. However, the types and concentrations of antibiotics at certain sites varied depending on the period, likely due to precipitation and the aforementioned amount of antibiotics. The concentration of antibiotics in the Han River was higher than that in the Yangtze River, primarily consisting of SMD used for the treatment of urinary tract infections, respiratory tract infections, tuberculosis, and other bacterial infections. This could be attributed to the dense population distribution along the Han River and the impact of medical wastewater discharged by nearby hospitals.

**Figure 2:**
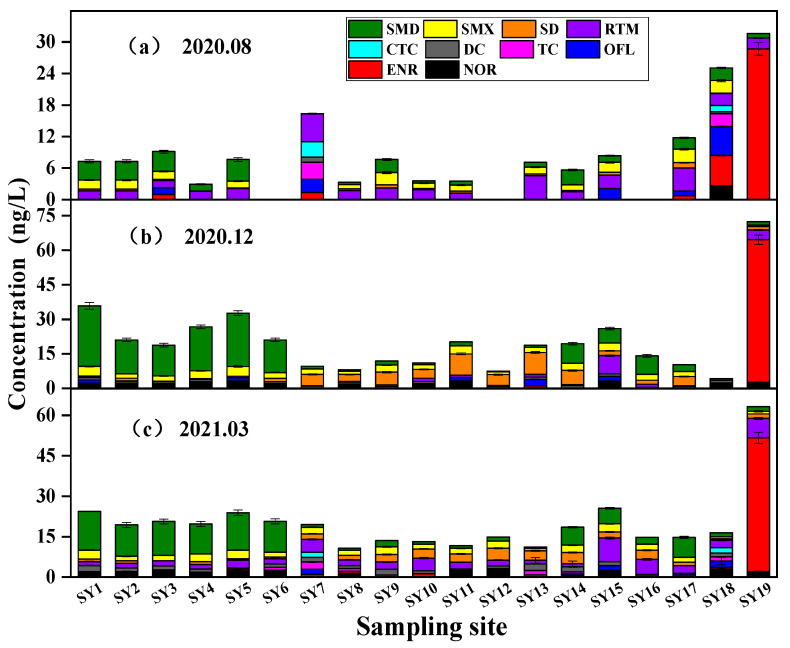
Distribution of target antibiotics in water source areas of Wuhan in (**a**) August 2020, (**b**) December 2020, and (**c**) March 2021.

Figure 2a depicts the variation in antibiotic types and concentrations at different sites during the wet season of the river. In general, the antibiotic levels in the target waters during the wet season ranged from 2.944 to 31.610 ng/L at each monitoring point. Among the different categories of antibiotics, SY18 exhibited the highest detection frequency, followed by SY3 and SY7. SY4 displayed the lowest concentration of antibiotics (2.944 ng/L), while SY19 exhibited the highest detection concentration (31.610 ng/L). Only SMD, ENR, and RTM were detected at SY19. However, the highest concentration of ENR was recorded at this site (28.686 ng/L). This can be attributed to the predominant use of ENR in animal husbandry, aquaculture, and other related industries, as the water intake is situated in an animal husbandry area. Figure 2b illustrates the range of total antibiotic concentrations at each site during the dry season, which varied from 4.285 to 72.455 ng/L. The lowest concentration at SY18 was noted during the dry season, while the highest concentration was observed at SY19, which is consistent with the wet season findings. During the dry season, a slightly greater variety of antibiotics were detected at each monitoring point compared to the wet season, with the exception of SY18, where five antibiotics were detected. Figure 2c shows that the total antibiotic concentration at each monitoring point during the normal water season ranged from 10.701 to 63.552 ng/L. The lowest detected concentration of total antibiotics in the normal water season was located at SY8, while the highest detected concentration remained at SY19, consistent with observations from both the wet and dry periods. The total concentration of antibiotics detected was similar across all sampling points, apart from SY19. With SY18 exhibiting the highest number of detected antibiotic types. This pattern could be attributed to the presence of numerous residential areas, farming areas, and agricultural irrigation sites along the SheShui, leading to the utilization of a diverse range of antibiotics. There were differences in the level of antibiotics at each monitoring point in the sampling area in the different periods; however, the highest values of the three antibiotics were consistently recorded at SY19. It is likely that the comparatively slow water flow of the SheShui River versus the Yangtze River and the Han River, coupled with the extensive agricultural and farming areas in its vicinity, contributed to the higher antibiotic concentrations.

### 3.2. Antibiotic Risk Assessment

This study employed the ECOSAR simulation method to calculate the toxicity values of 15 antibiotics detected in the surface water bodies of drinking water sources in Wuhan. The toxicity values for fish, daphnia, and green algae were categorized into four groups: highly toxic, toxic, harmful, and harmless. As indicated in Table 4, among the fifteen antibiotic pollutants, eight (53.33%) were classified as toxic and harmful. The chronic toxicity of these eight antibiotics exhibited strong toxicity towards daphnia and was highly toxic to fish and green algae. The acute toxicity of ETM and RTM was harmful to fish, while the other two types were considered toxic and harmful. Their chronic toxicity towards fish and green algae was classified as toxic. The remaining seven compounds were deemed harmless, but the chronic toxicity of ENR, TC, CTC, and DC was harmful to daphnia.

To quantify the toxicity of residual antibiotics in the aquatic environment to organisms in aquatic ecosystems, the RQs model was used to evaluate the potential risks of pollutant residues in the water bodies [43,44]. Fish, water fleas, and green algae were utilized to represent the three distinct nutritional levels of vertebrates, invertebrates, and phototrophs, respectively. Table 5 illustrates that the majority of antibiotics had minimal or no detrimental effects on the aquatic organisms in the water sources (RQ < 0.1). SMX and SD consistently posed a moderate risk to Daphnia (0.1 < RQ < 1); SMD in the Yangtze River, JuShui, and SheShui presented a moderate risk to Daphnia, while SMD from the Han River posed a high risk to Daphnia (RQ = 2.41). The ENR of JuShui indicated a low risk to Daphnia. Apart from the moderate risk to Daphnia in JuShui, RTM demonstrated a low risk to fish, Daphnia, and green algae in the other rivers. The current evaluation method does not take into account the potential interactions between multiple compounds that may result in synergistic effects, and the available toxicological data are limited; therefore, further research on the influencing factors is necessary.

### 3.3. Spatial and Temporal Distribution of ARGs

**Figure 3:**
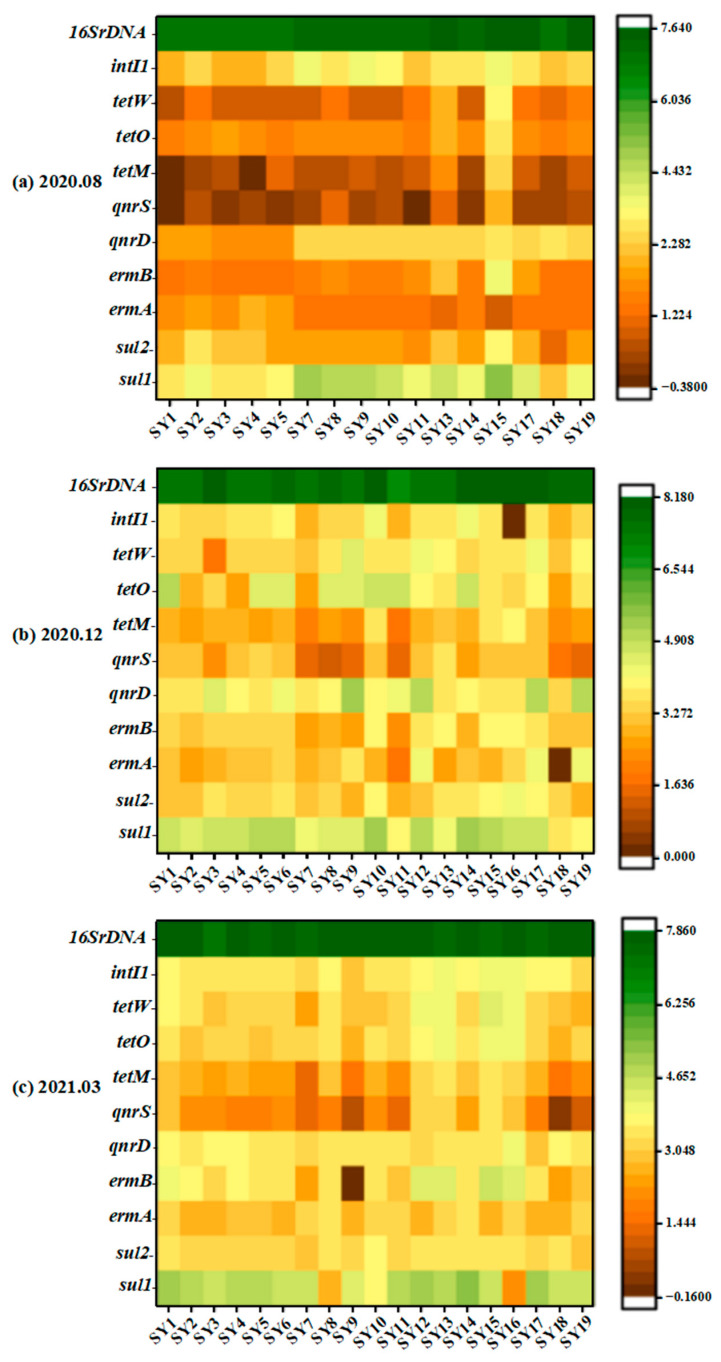
Absolute quantitative logarithmic heat map of ARGs at various sampling points in different periods: (**a**) August 2020, (**b**) December 2020, and (**c**) March 2021. Each grid represents the relative abundance of the target genes for all samples normalized in a log 10 scale. The horizontal axis represents the sequence of sampling sites, while the vertical axis represents the target genes.

Three surface water samples were collected from water sources in Wuhan and tested for 11 genes including *sul1*, *sul2*, *tetM*, *tetO*, *tetW*, *qnrS*, *qnrD*, *ermA*, *ermB*, integron *intI1*, and the reference gene 16S rDNA. The target gene was detected in all water samples; the absolute abundance was in the range of 4.36 × 10^−1^~1.50 × 10^8^ copies/mL, and the absolute range of ARGs in water samples during the wet season was 4.36 × 10^−1^~4.32 × 10^7^ copies/mL. The absolute abundance range of ARGs during the dry season was 2.26 × 10^1^~1.50 × 10^8^ copies/mL, and the absolute abundance range of ARGs during the normal water season was 7.11 × 10^−1^~7.05 × 10^7^ copies/mL. The heat map of resistance genes drawn from the logarithm of the absolute abundance of resistance genes determined by three samples is shown in Figure 3. The main types of ARGs in the water bodies in this sampling area were *sul1*, *qnrD*, and *tetO*. The study of resistance genes in the surface water of the lower Yangtze River also yielded similar results [30]. Among the nine ARGs analyzed, *sul1* demonstrated the highest absolute abundance in the water samples. This could be attributed to the inherent stability of sulfonamide resistance genes in the environment. These ARGs are readily acquired by *intI1* and consistently disseminate in the environment, suggesting that the abundance of these genes should be expected to gradually increase over time [30,35].

**Figure 4:**
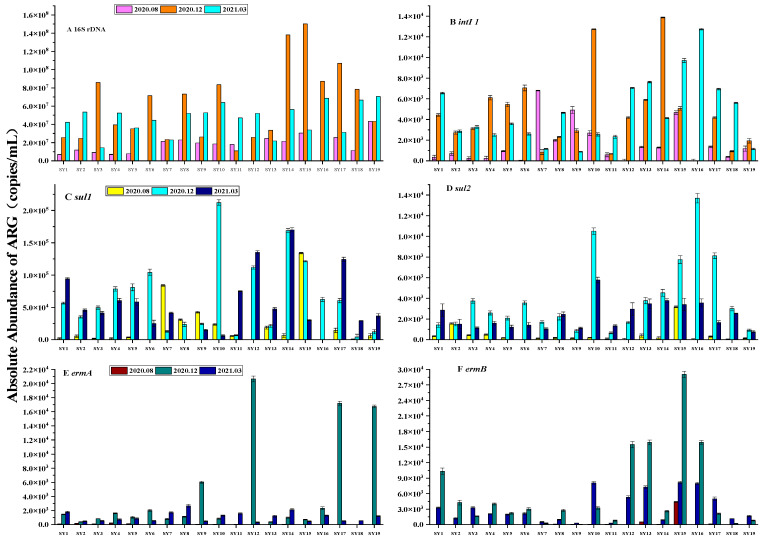

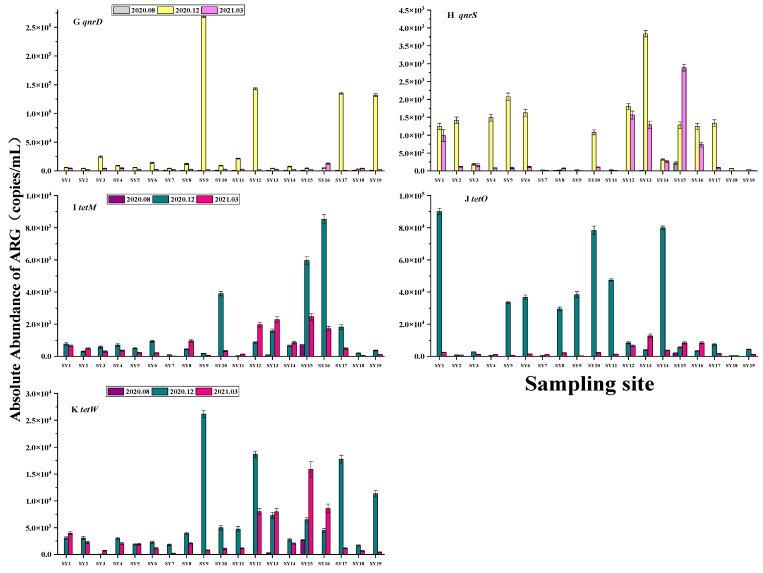
Gene abundance measured in surface water collected from various water sources during different periods from August 2020 to March 2021. (**A**) 16S rRNA, (**B**) *intI1*, (**C**) *sul1*, (**D**) *sul2*, (**E**) *ermA*, (**F**) *ermB*, (**G**) *qnrD*, (**H**) *qnrS*, (**I**) *tetM*, (**J**) *tetO*, (**K**) *tetW*.

**Figure 5:**
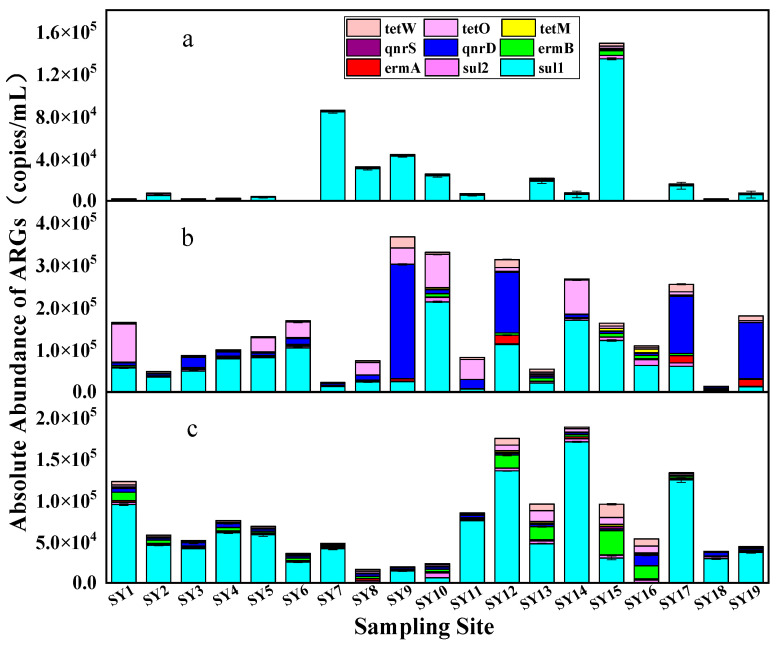
Distribution of various ARGs at various sampling points in different periods (**a**–**c** are the high, low, and normal water seasons, respectively).

The absolute abundance values of sulfonamide resistance genes were generally higher in the dry and normal water seasons and relatively low in the wet season, especially for *sul2* (Figure 4). *sul1* was the predominant gene in all samples. The absolute abundance in the wet season ranged from 6.40 × 10^2^ to 1.34 × 10^5^ copies/mL, in the dry season from 4.13 × 10^3^ to 2.12 × 10^5^ copies/mL, and in the normal water season from approximately 1.32 × 10^2^ to 1.35 × 10^5^ copies/mL (Figure 4). For *sul2*, the average absolute abundances in the corresponding periods were 4.90 × 10^2^, 3.91 × 10^3^, and 2.29 × 10^3^ copies/mL, respectively. The detection rates of *ermA* and *ermB* were both 100%. The average absolute abundance values of *ermA* and *ermB* during the dry period were 3.005 × 10^3^ and 3.309 × 10^3^ copies/mL, respectively, which were higher than the normal water and wet seasons (2.950 × 10^3^ and 3.287 × 10^3^ copies/mL and 1.549 × 10^3^ and 1.759 × 10^3^ copies/mL, respectively). Figure 4 illustrates that the measured values of *ermB* were slightly higher than *ermA* for the samples collected during the same period, with the exception of specific points (SY12, SY17, and SY19). However, the overall difference is not statistically significant. The difference in abundance values between *qnrD* and *qnrS* is more pronounced. *qnrD* exhibited an absolute advantage compared to *qnrS* (Figure 4). In terms of the quinolone genes, the overall abundance of the two genes was relatively evident. The absolute abundance of the two genes was as follows: dry season > normal water season > wet season. The absolute abundance of tetracycline resistance genes followed the trend of *tetO* > *tetW* > *tetM* (Figure 5), and the absolute abundance values of tetracycline and quinolone resistance genes exhibited the same temporal distribution difference.

Furthermore, the detection of *intI1* in the study area was more prevalent, and the absolute abundance value of *intI1* varied significantly in each period (2.27 × 10^2^~6.80 × 10^3^ copies/mL in the wet season, 6.75 × 10^2^~1.27 × 10^4^ copies/mL in the dry season, and 8.79 × 10^2^~1.27 × 10^4^ copies/mL in the normal water season). Previous research has indicated that a type of integron *intI1* can enhance the likelihood of horizontal transfer of ARGs, especially sulfonamide resistance genes [45,46,47]. Therefore, the distribution of *intI1* is similar to that of sulfonamide genes, and this explains one of the possible reasons why sulfonamide genes are significantly higher than other types of ARGs. The abundance of ARGs at each sampling site during different time seasons is depicted in Figure 4. It can be seen from Figure 4 that, in comparison to the Yangtze River, although the types and absolute monthly abundances of resistance genes at S1~S6 on the Hanjiang River exhibited variation, the differences between each site were not significant. In the wet season, the ARGs of the sampling points of the Yangtze River were higher than those of the tributaries, with *sul1* having an absolute advantage at each point. The absolute abundances of total resistance genes measured at sampling points on the Yangtze River during the dry season were higher than that of the Han River (the average absolute abundance values of ARGs of the Yangtze River, Han River, SheShui, and JuShui were 2.05 × 10^4^, 1.29 × 10^4^, 1.51 × 10^3^, and 1.99 × 10^4^ copies/mL, respectively). The absolute abundances of ARGs at seven sites, excluding SY7, SY8, and SY10, were higher than those of the Han River, and *sul1*, *qnrD*, and *tetO* were the dominant genes. The general trend of the river in the normal water season was similar to that in the dry season. The average absolute abundance of the total ARGs was Yangtze River > Han River > JuShui > SheShui, and *sul1* occupied an absolute advantage.

Figure 4 shows that the absolute abundances of ARGs on the Yangtze River at the sampling points in the urban area were higher than those in the suburbs. This observation can be attributed to the dense urban population and the high flow of people, leading to more pollutant emissions. Notably, the highest absolute abundance of ARGs was observed at SY9 during the dry season. This could be attributed to the presence of multiple sewage treatment plants in close proximity to this sampling point, with one plant located upstream. Additionally, human resistance to antibiotics may be enhanced during the winter months due to the escalated use of antibiotics. Consequently, this may result in a higher concentration of antibiotics. The total abundance at SY12 during the dry and normal water seasons was higher than that at SY11. This may be because there are more large hospitals around SY12 or because it is close to the confluence of the Han and Yangtze Rivers, which has a low detection result value. The gene abundances at SY18 and SY19 in SheShui and JuShui were not high. The total ARGs measured at SY18 in the wet and dry seasons were considerably low, which indicates that the location of this point in the river is less likely to be polluted by ARGs than other sites.

In summary, the absolute abundance levels of sulfonamide, tetracyclic, and macrolide ARGs in the surface water of the water source of Wuhan City were relatively high, while the level of quinolones was low. It can be seen from Figure 5 that the sequence of ARG concentration at different sampling points during the wet season was SAs > FQs > TCs > MLs. The specific performance of various ARGs at different sampling points during the dry season was *sul1* > *qnrD* > *tetO* > *tetW* > *ermA* > *sul2* > *ermB* > *tetW* > *qnrS*. The general trend of ARGs at each sampling point in the normal water season was consistent with that in the wet and dry seasons.

### 3.4. Correlation between ARGs and Antibiotics

**Figure 6:**
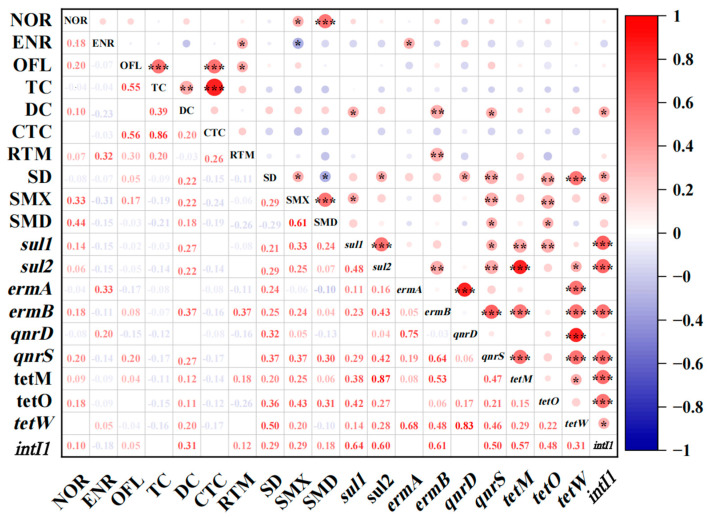
Correlations among antibiotics and ARGs. Red represents positive correlation, and blue represents negative correlation. * represents a significant relationship, ** represents a tight relationship, and *** represents an extremely tight relationship.

The Pearson correlation coefficient was utilized in SPSS to analyze the correlation between ARGs and antibiotics. Conventional water quality indicators were also used. Moreover, a heat map was used to preliminarily assess the presence of a relationship between the two variables (* denotes a relationship between the two, blank means there is no relationship). The results of the correlation analysis between ARGs and antibiotics are presented in Figure 6 and Supplementary Table S7.

It is evident from Figure 6 and Supplementary Table S7 that the majority of ARGs did not exhibit a strong correlation with their corresponding antibiotics, but rather showed a close relationship with other types of antibiotics. In the study area, *sul1* and DC; *ermB* and DC; *qnrS*, *tetO*, and SMX; and *tetW* and SD demonstrated close relationships with positive correlations (*p* < 0.01), and the correlation coefficient values were 0.460, 0.568, 0.427, 0.506, 0.527, and 0.528, respectively. In addition, *ermB* and SMX; *qnrS* and DC; SD and SMD; *tetO* and SD; SMD, *sul1*, and DC; SMX, *ermA*, and ENR; and *qnrD* and SD also showed correlations (*p* < 0.05). The induction of ARGs is influenced by other types of antibiotics, which can induce the production of non-corresponding ARGs, and a single antibiotic may trigger the production of multiple ARGs. All correlations between ARGs and antibiotics in this study area were positive. However, contrary to these findings, other studies have reported a negative correlation between ARGs and antibiotics [44], possibly due to the more complex influencing factors in the water environment. Moreover, the map indicated a significant correlation between *intI1* and *sul1*, *sul2*, *ermB*, *qnrS*, *tetM*, *tetO*, and *tetW*, suggesting that *intI1* may promote the proliferation of these ARGs. Therefore, it is believed that the proliferation of ARGs could increase their potential for transmission through integron increase. Previous studies have also confirmed that *intI1* contributes to the spread of ARGs [48,49]. Besides the direct influence of antibiotics, there are additional environmental pressures that also contribute to the formation of co-selection and cross-selection effects.

## 4. Conclusions

In this paper, the occurrence and the temporal and spatial distributions of antibiotics and ARGs in drinking water sources including the Yangtze River, Han River and other rivers in Wuhan City and their correlations were investigated. The observed trend in the temporal distribution was mainly dry season > normal season > wet season; the spatial distribution trend was JuShui > SheShui > Han River > Yangtze River. The ecological risk assessment of antibiotics showed that most antibiotics pose no or very little risk to aquatic organisms in water sources, but SD, SMX, and SMD showed medium and high risks to Daphnia, and the supervision of the use and discharge of these antibiotics should be strengthened. The distribution of antibiotics and ARGs varied by sampling time and location. *sul1*, *qnrD*, and *tetO* showed a high abundance in all the samples. Correlation analysis found that the distributions of most ARGs correlated weakly with the corresponding antibiotics, but were strongly associated with other types of the antibiotics. This study was able to demonstrate the main patterns of antibiotic as well as resistance gene contamination in the Wuhan section of the Yangtze River in a more systematic way, to better understand the sources of antibiotics in the environment, to maintain the safety of drinking water in the region, and to provide valuable insights and data on antibiotic resistance and water contamination issues of concern in other regions. However, the interference of other kinds of pollutants in the water body and the migration transformation of pollutants in the sediments on the target pollutants in the overlying water was not taken into account in the research process, and at the same time, due to the structural instability of some antibiotics and ARGs, their concentration determination values may not have been completely accurate, which may have had certain impacts, and this needs to be further explored in subsequent research.

## Figures and Tables

**Table 1:** Physicochemical properties of target antibiotics.

Category	Compound	Abbreviation	Molecular Weight	Molecular Formula	Internal Standard
SAs	Sulfamethazine	SMZ	278.33	C_12_H_14_N_4_O_2_S	Sulfamethazine-^13^C_6_ Sulfamethoxazole-d_4_
	Sulfadiazine	SD	250.28	C_10_H_10_N_4_O_2_S	
	Sulfapyridine	SP	249.29	C_11_H_11_N_3_O_2_S	
	Sulfamethoxazole	SMX	253.27	C_10_H_11_N_3_O_3_S	
	Sulfameter	SMD	280.3	C_11_H_12_N_4_O_3_S	
	Sulfamerazine	SM	264.30	C_11_H_12_N_4_O_2_S	
FQs	Norfloxacin	NOR	319.33	C_16_H_18_FN_3_O_3_	Ciprofloxacin-d_8_
	Ofloxacin	OFL	331.34	C_18_H_20_FN_3_O_4_	
	Enrofloxacin	ENR	359.4	C_19_H_22_FN_3_O_3_	
TCs	Tetracycline	TC	444.43	C_22_H_24_N_2_O_8_	Thiabendazole-d_4_
	Oxytetracycline	OTC	496.90	C_22_H_28_N_2_O_11_	
	Chlorotetracycline	CTC	515.34	C_22_H_23_ClN_2_O_8_	
	Doxycyclinehyclate	DC	444.44	C_22_H_24_N_2_O_8_	
MLs	Erythromycin	ETM	731.95	C_38_H_69_NO_12_	Erythromycin-^13^C-d_3_
	Roxithromycin	RTM	837.05	C_41_H_76_N_2_O_15_	

**Table 2:** Detailed information of target ARGs.

Category	Nomenclature
Tetracycline	*tetM*
	*tetO*
	*tetW*
Quinolone	*qnrS*
	*qnrD*
Sulfa	*sul1*
	*sul2*
Cyclolactone	*ermA*
	*ermB*
Bacterial reference gene	*16S rDNA*
I integrons	*intI1*

**Table 3:** Summary of the occurrence of various antibiotics in different periods (ng/L).

Antibiotic Name	August 2020 (Wet Season)			December 2020 (Dry Season)			March 2021 (Normal Water Season)		
	The Detection Rate %	Average ng/L	Max ng/L	The Detection Rate %	Average ng/L	Max ng/L	The Detection Rate %	Average ng/L	Max ng/L
SMD	93.75	1.871	4.110	100	7.685	26.317	94.74	5.738	14.357
SMX	75	1.597	2.566	94.74	2.576	4.190	100	2.148	3.361
SD	75	0.361	1.008	73.68	4.202	9.331	94.97	2.123	4.373
RTM	100	2.381	5.302	94.74	1.217	7.903	100	3.167	8.746
ENR	31.25	7.509	28.686	73.68	4.633	61.883	73.68	4.184	49.883
NOR	6.25	2.65	2.65	63.16	2.376	3.082	63.16	2.157	2.965
OFL	31.25	2.448	5.472	26.32	1.840	3.415	42.11	1.147	2.331
DC	12.50	0.699	1.002	78.95	0.917	1.128	73.68	1.538	2.406
TC	12.50	2.852	3.260	0	—	—	36.84	1.389	2.762
CTC	12.50	2.066	2.968	0	—	—	10.53	1.970	2.010

**Table 4:** Toxicity values of major antibiotics in rivers calculated based on ECOSAR.

Compound	CAS	Acute Toxicity Value (ng/L)			Chronic Toxicity Value (ng/L)			Compound Toxicity
		Fish (LC50)	Daphnia (LC50)	Algae (EC50)	Fish (ChV)	Daphnia (ChV)	Algae (ChV)	
SD	68-35-9	907	10.3	40.4	23.8	0.101	29	Harmful
SP	144-83-2	246	6.17	20.8	4.54	0.066	10.4	Toxic
SMX	723-46-6	267	6.43	21.8	5	0.068	11.1	Toxic
SMD	651-06-9	899	11.1	42.5	22.9	0.109	29.5	Harmful
SM	127-79-7	421	7.89	28.1	8.83	0.082	16.1	Toxic
SMZ	57-68-1	195	6.02	15.9	3.26	0.065	8.88	Toxic
OFL	82419-36-1	19,400	1790	2440	2460	114	675	Non-toxic
NOR	70458-96-7	20,100	1830	2570	2650	116	703	Non-toxic
ENR	93106-60-6	4920	505	561	454	35.8	167	Non-toxic
TC	60-54-8	13,100	1060	1890	2490	59.9	474	Non-toxic
OTC	79-57-2	139,000	9440	23,800	45,500	447	5270	Non-toxic
CTC	57-62-5	5340	446	712	804	28.2	189	Non-toxic
DC	564-25-0	13,900	1120	2000	2660	62.7	501	Non-toxic
ETM	114-07-8	68.4	8.62	6.37	3.36	0.747	2.2	Harmful
RTM	80214-83-1	51.6	6.72	4.66	2.3	0.601	1.65	Harmful

Note: According to GHS regulations: strong toxicity (LC50/EC50/ChV < 1, yellow), toxic (1 < LC50/EC50/ChV < 10, green), harmful (10 < LC50/EC50/ChV < 100, blue) and harmless (LC50/EC50/ChV > 100, colorless). Hazard categories are determined based on the lowest acute toxicity of compounds to different species of aquatic organisms.

**Table 5:** Risk assessment of the target antibiotics to sensitive aquatic organisms.

	RQ (The Han River)			RQ (The Yangtze River)			RQ (SheShui)			RQ (JuShui)		
	Fish	Daphnia	Algae	Fish	Daphnia	Algae	Fish	Daphnia	Algae	Fish	Daphnia	Algae
SD												
SMX												
SMD												
OFL												
NOR												
ENR												
TC												
CTC												
DC												
RTM												

Note: Green denotes no risk, RQ ≤ 0.01; yellow denotes low risk, 0.01 ≤ RQ < 0.10; blue denotes medium risk, 0.1 ≤ RQ < 1.00; red denotes high risk, RQ > 1.00.

## Data Availability

Data are contained within the article.

## Supplementary Materials

The following supporting information can be downloaded at: https://www.mdpi.com/article/10.3390/toxics12070507/s1, Text S1: Standards and chemicals; Text S2: Details of the sampling points; Text S3: Antibiotic sample pretreatment and analysis; Text S4: ARGs sample pretreatment and analysis; Figure S1: Concentration distribution of various antibiotics at various sampling points in different periods; Figure S2: Concentration distribution of conventional pollutants at various sampling points in different periods; Table S1: Sites and physicochemical parameters of samples; Table S2: Steps and parameters of solid phase extraction; Table S3: Instrument gradient elution program; Table S4: UPLC–MS/MS determination of antibiotic mass spectrometry conditions MS/MS; Table S5: Detection limit, quantification limit, and linear range of antibiotics; Table S6: Primers used for quantification of ARGs; Table S7 Correlation coefficient statistics table of antibiotics and ARGs.
