# Supplementary material for: Study on the Distribution Characteristics and Risk Assessment of Antibiotics and Resistance Genes in Water Sources of Wuhan
Source: Toxics. 2024 Jul 14;12(7):507. doi: 10.3390/toxics12070507 (PMC11280968; doi:10.3390/toxics12070507)
Supplement: Supplementary file 1 [file toxics-12-00507-s001.zip › toxics-3083206-supplementary.pdf]

Supplementary data

Figure captions:

Figure S1 Concentration distribution of various antibiotics at various sampling points in different periods.

Figure S2 Concentration distribution of conventional pollutants at various sampling points in different periods.

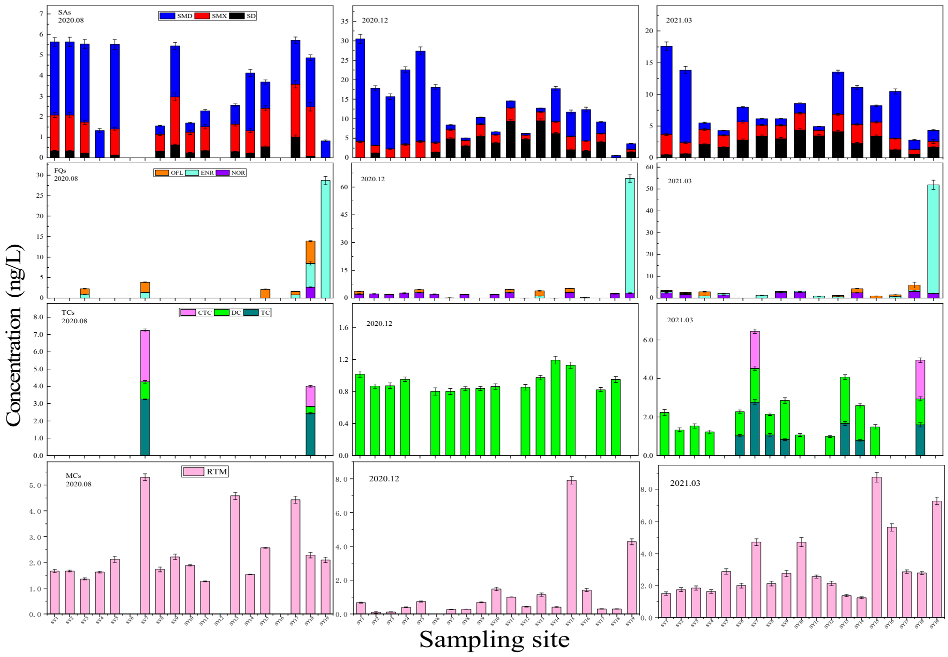

Figure S1 Concentration distribution of various antibiotics at various sampling points in different periods

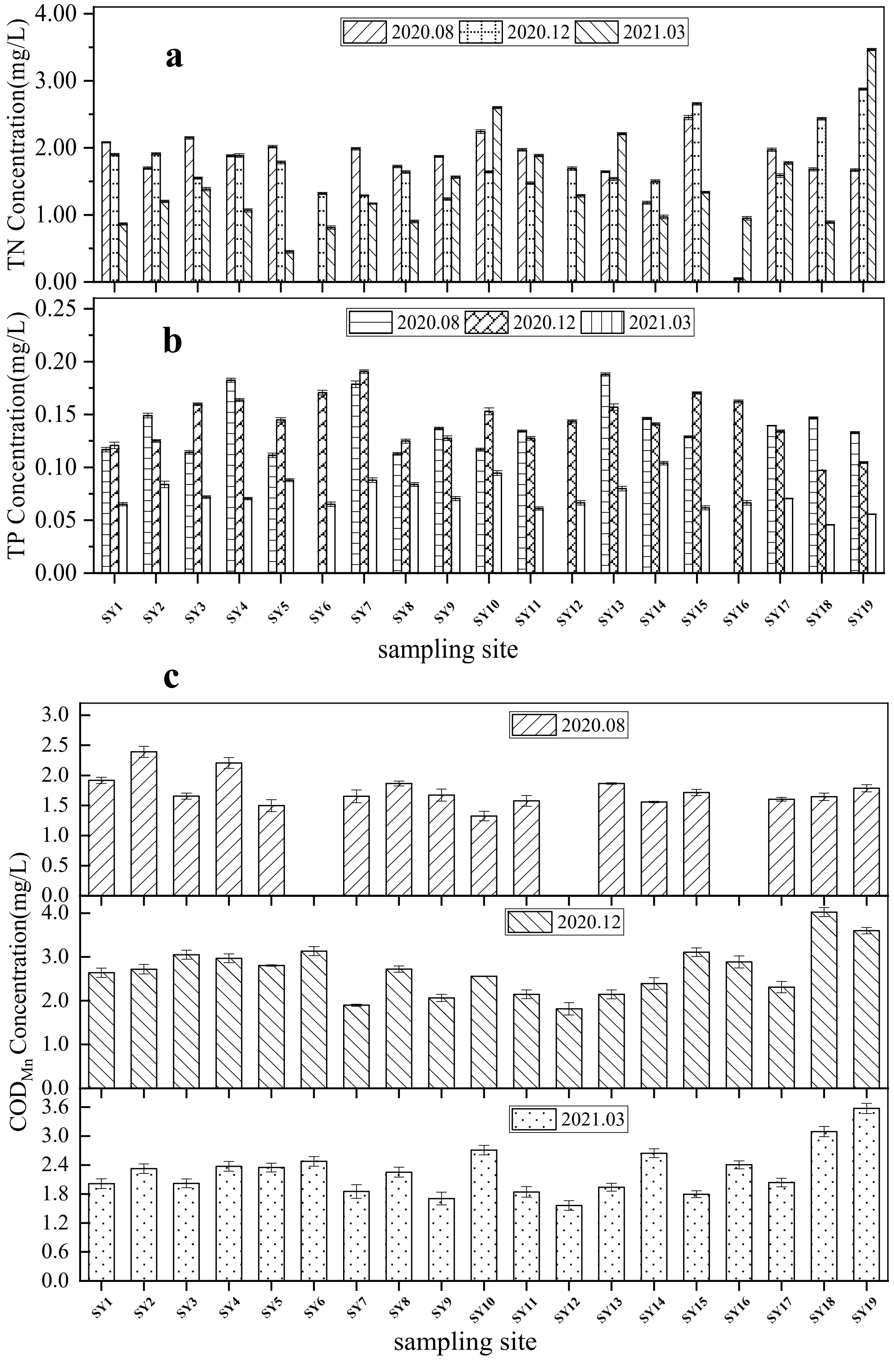

Figure S2 Concentration distribution of conventional pollutants at various sampling points in different periods. a, TN; b, TP; c, COD_{Mn}.

Text S1 Standards and chemicals

Fifteen antibiotics: sulfonamides (SAs): sulfapyridine (SP), sulfadiazine (SD), sulfamethoxazole (SMX), sulfamethazine (SMZ), sulfamethazine (SM), and sulfamethoxazole (SMD); tetracyclines (TCs): tetracycline (TC), doxycycline (DC), oxytetracycline (OTC), and chlortetracycline (CTC); Quinolones (FQs): Norfloxacin (NOR), ofloxacin (OFL), and enrofloxacin (ENR); and macrolide (MCs): roxithromycin (RTM) and erythromycin (ETM) were purchased from Dr Ehrenstorfer, Germany. Sulfamethoxazole-d4 (SMX-d4), sulfamethazine-$^{13}C_6$ (SMZ-$^{13}C_6$), thiabendazole-d4 (TBZ-d4), ciprofloxacin-d8 (CPF-d8), erythromycin-^{13}C, d3 (ETM-^{13}C, d3), and other substitutes were purchased from Toronto Chemical Company, Canada. The internal standard atrazine-$^{13}C_3$ (atrazine-$^{13}C_3$) was purchased from Dr Ehrenstorfer, Germany. The solid phase extraction column (500 mg, 6 mL) was purchased from Waters, USA. Chromatographic pure reagents such as methanol, acetonitrile, and formic acid were purchased from Thermo Fisher Scientific (China) Co., Ltd. All test water was Millipore water.

This study selected 11 ARGs as target genes, including tetracycline (*tetM*, *tetO*, and *tetW*), quinolone (*qnrS* and *qnrD*), sulfa (*sul1* and *sul2*), and Cyclolactone (*ermA* and *ermB*) antibiotic resistance genes. In addition, 16S rDNA was selected as the bacterial reference gene. Since the presence of type I integrons may impact the spread of resistance genes, the test for the integron intIl was added to the experiment.

Text S2 Details of the sampling points

Taking the drinking water sources in Wuhan as the research object, 19 sampling points were selected. The specific locations are shown in Fig. 1. Among them, S1~S6 are located in the Hanjiang River, S7~S17 are located in the Yangtze River, and S18~S19 are located in Sheshui and Jushui. Supplementary Table S1 shows details of

the sampling points, including longitude and latitude, some conventional water quality indicators.

Text S3 Antibiotic sample pretreatment and analysis

The solid phase extraction method was used to enrich the antibiotics in the water sample. The 1 L water sample was filtered through an aqueous filter (0.45 μm), and then extracted through a pretreated Oasis HLB cartridge (Waters, USA) on a fully automatic solid phase extraction (SPE) device. Antibiotics were analyzed using ultra performance liquid chromatography-tandem mass spectrometry (UPLC-MS/MS) (Waters, USA) equipped with ACQUITY UPLC BEH C18 (2.1 × 100 mm, 1.7 μm) and ionized in ESI (+) mode. During sample analysis, the chromatographic column temperature was 40 °C, which comprised mobile phases A (0.1% formic acid water) and B (0.1% formic acid acetonitrile), and a gradient elution procedure was used to achieve target separation. The antibiotic concentration was quantified using an internal standard and showed a good linear relationship ($r > 0.99$) in the linear range of 0.5~50 μg/L. The detection limit of the target antibiotic method was 0.04~0.09 ng/L (S/N = 3), and the limit of quantification was 0.16~0.36 ng/L (S/N = 10). The target antibiotic recovery rate ranged from 59.5% to 102.8%, and the relative standard deviation (RSD) did not exceed 11.2% ($n = 6$). The details of sample solid phase extraction parameters, instrument gradient elution program, mass spectrometry information, regression equation, and linear range are shown in Table S2~S5.

Text S4 ARGs sample pretreatment and analysis

The vacuum filtration method was used to enrich the microorganisms in the water sample. First, 500 mL of the water sample was filtered through a 0.45 μm filter membrane, the filter membrane was collected and cut into PowerBead Tubes provided by the DNA extraction kit, and the sample was stored at -80 °C before performing DNA

extraction and analysis. In the experiment, the PowerSoil@DNA Isolation kit was used to extract DNA. A trace nucleic acid protein analyzer Nanodrop2000 (Thermo Scientific, USA) was used to detect the DNA content and purity. The change in fluorescence signal was used to detect the change in the amount of amplified product in each cycle of the PCR amplification reaction in real time, and the starting template was quantitatively analyzed via the relationship between the Ct value and the standard curve. Spot samples were synthesized in triplicate, and sterile water was used as a negative control. The calibration curve showed a good linear relationship (R2>0.99). The PCR amplification efficiency ranged from 95% to 110%, indicating that the efficiency of qPCR meets the requirements. Details of primers can be found in the Table S6.

Table captions:

Table S1 Sites and physicochemical parameters of samples.

Table S2 Steps and parameters of solid phase extraction.

Table S3 Instrument gradient elution program.

Table S4 UPLC-MS/MS determination of antibiotic mass spectrometry conditions MS/MS

Table S5 Detection limit, quantification limit, and linear range of antibiotics.

Table S6 Primers used for quantification of *ARGs.*

Table S7 Correlation coefficient statistics table of antibiotics and ARGs.

Table S1 Sites and physicochemical parameters of samples

Sample ID	Longitude and Latitude	Temperature (℃)			pH			TN (mg/L)			TP (mg/L)			COD_{Mn} (mg/L)		
		2020.08	2020.12	2021.03	2020.08	2020.12	2021.03	2020.08	2020.12	2021.03	2020.08	2020.12	2021.03	2020.08	2020.12	2021.03
S1	113.966632E, 30.67968N	28.4	8.8	11.5	8.15	7.99	8.08	2.082	1.899	0.865	0.117	0.121	0.055	28.400	2.639	2.012
S2	114.030966E, 30.592072N	28.5	9.0	11.4	7.99	7.95	7.94	1.697	1.910	1.202	0.149	0.125	0.074	35.403	2.720	2.325
S3	114.120144E, 30.596426N	28.7	9.5	11.7	7.89	7.97	8.06	2.149	1.548	1.384	0.114	0.160	0.062	24.510	3.052	2.020
S4	114.138894E, 30.592189N	28.7	10.0	11.8	7.98	7.88	8.02	1.881	1.884	1.068	0.182	0.164	0.060	32.679	2.969	2.373
S5	114.187260E, 30.581992N	28.9	9.8	11.7	8.06	8.01	8.04	2.016	1.783	0.451	0.111	0.145	0.078	22.175	2.804	2.349
S6	114.221506E, 30.574307N	-	9.4	11.6	-	7.99	8.07	-	1.319	0.812	-	0.231	0.055	-	3.134	2.477
S7	114.095008E, 30.312724N	29.6	12.5	12.3	8.05	8.11	8.06	1.988	1.288	1.169	0.179	0.190	0.078	24.467	1.897	1.852
S8	114.120623E, 30.337628N	29.1	10.6	12.1	7.87	8.06	7.90	1.723	1.640	0.902	0.113	0.125	0.074	27.622	2.722	2.253
S9	114.155159E, 30.383538N	29.6	12.4	12.1	8.04	8.09	7.70	1.873	1.237	1.563	0.137	0.127	0.060	24.759	2.062	1.707
S10	114.216957E, 30.470277N	29.4	11.6	12.4	7.88	8.03	7.92	2.242	1.642	4.800	0.117	0.153	0.085	6.614	2.557	2.709
S11	114.234810E, 30.472940N	29.5	11.9	12.1	8.05	8.08	7.94	1.971	1.473	1.885	0.134	0.127	0.051	23.342	2.144	1.844
S12	114.280043E, 30.555387N	-	12.1	12.5	-	8.07	7.85	-	1.691	1.287	-	0.144	0.056	-	1.814	1.563
S13	114.322185E, 30.600980N	29.5	11.6	12.5	7.77	8.10	7.73	1.644	1.537	2.210	0.188	0.157	0.070	27.622	2.144	1.940
S14	114.355659E, 30.658882N	29.5	11.6	12.2	7.88	8.05	8.03	1.182	1.501	0.969	0.146	0.141	0.094	5.058	2.392	2.645
S15	114.382095E, 30.648546N	29.2	10.7	12.3	7.89	7.91	7.86	2.452	3.158	1.338	0.129	0.170	0.042	7.392	5.110	1.796
S16	114.409904E, 30.665085N	-	11.1	12.6	-	8.08	8.23	-	0.050	0.949	-	0.162	0.056	-	2.887	2.405
S17	114.561310E, 30.635549N	29.3	11.5	12.1	8.06	8.07	8.19	1.971	1.589	1.774	0.140	0.134	0.060	23.731	2.309	2.036
S18	114.385185E, 30.889557N	29.1	8.8	11.1	7.95	7.95	8.07	1.679	2.434	0.892	0.147	0.067	0.026	24.365	4.124	3.094
S19	114.787474E, 30.852132N	29.1	8.5	12.0	8.05	8.09	8.03	1.667	2.876	3.467	0.133	0.085	0.046	26.455	3.299	4.072

Table S2 Steps and parameters of solid phase extraction

Step	Solvent	Volume (mL)	Flow rate (mL/min)	Collect	Dry (s)
Activation	Methanol	10	3	Waste 1	25
Activation	Water	10	3	Waste 2	35
Needle washing	Water	/	/	Waste 2	10
Sample loading	/	1000	10	Waste 2	35
Needle washing	Water	/	/	Waste 2	10
Rinse	Water	10	3	Waste 2	35
Elution	Methanol	10	0.5	Component 1	35

Table S3 Instrument gradient elution program

Time (min)	Flow rate (mL/min)	Mobile phase A (%)	Mobile phase B (%)
0	0.3	95	5
5	0.3	70	30
11	0.3	40	60
13	0.3	10	90
16	0.3	10	90
16.5	0.3	95	5
22	0.3	95	5

Table S4 UPLC-MS/MS determination of antibiotic mass spectrometry conditions MS/MS

Category	Compound	Precursor ion (m/z)	Product ions (m/z)	Cone voltage (v)	Collision voltage(v)
Internal standard	atrazine-13C3	279.1	177.1，98.0	33	26，15
SAs	SD	251.3	92.2，156.2	30	25，15
	SP	250.3	108.2，156.2	33	22，15
	SMX	254.3	92.2，156.2	30	25，15
	SMZ	279.3	156.2，186.2	35	26，25
	SMD	281.3	92.2，156.2	35	25，15
	SM	265.3	156.2，172.2	35	15，15
	SMX-d4	258.2	112.2，160.1	30	22，15
	SMZ-13C6	285.2	124.2，186.1	30	22，15
TCs	TC	445.4	410.4，427.3	40	15，12
	OTC	461.5	201.3，426.3	20	32，18
	CTC	479.2	154.1，444.2	35	25，20
	DC	445.2	154.1，428.2	30	28，20
	TBZ-d4	206.1	135.1，179.1	30	30，25
FQs	NFC	320.4	233.3，302.3	40	25，17
	OFC	362.4	261.3，318.4	25	30，20
	EFC	360.4	316.4，342.3	38	18，23
	CPF-d8	340.2	296.2，322.2	20	20，20
MCs	ETM	734.9	158.3，576.6	30	30，20
	RTM	837.9	158.3，679.7	43	35，20
	ETM-13C，d3	739.0	120.2，162.2	28	30

Table S5 Detection limit, quantification limit, and linear range of antibiotics

Category	Compound	Regression curve	R	MDL (ng/L)	MQL (ng/L)	Linear range/(μg/L)
SAs	SD	A=0.0554696C+0.00128077	0.9998	0.04	0.16	0.5~50
	SP	A=0.10523C+0.0761165	0.9997	0.04	0.16	0.5~50
	SMX	A=0.120052C-0.0202906	0.9998	0.04	0.16	0.5~50
	SMZ	A=0.043639C+0.00489198	0.9997	0.04	0.16	0.5~50
	SMD	A=0.105042C+0.0354494	0.9998	0.04	0.16	0.5~50
	SM	A=0.0890275C+0.00453012	0.9992	0.04	0.16	0.5~50
TCs	TC	A=0.0594875C-0.0188063	0.9980	0.06	0.24	0.5~50
	OTC	A=0.0544099C-0.00482624	0.9988	0.08	0.32	0.5~50
	CTC	A=0.0246718C-0.00417718	0.9992	0.09	0.36	0.5~50
	DC	A=0.0508174C+0.00176639	0.9994	0.08	0.32	0.5~50
FQs	NFC	A=0.0383776C+0.0335269	0.9997	0.06	0.24	0.5~50
	OFC	A=0.0926263C-0.03211833	0.9970	0.06	0.24	0.5~50
	EFC	A=0.143444C+0.0379489	0.9989	0.07	0.28	0.5~50
MCs	ETM	A=0.00561921-0.00111531	0.9998	0.05	0.20	0.5~50
	RTM	A=0.0873188C+0.0238989	0.9996	0.05	0.20	0.5~50

Table S6 Primers used for quantification of ARGs

Sort	Gene	Primer	Sequence (5'-3')	Amplicon Length (bp)	Annealing temperature (℃)
1	*qnrS*	*qnrS-F*<br>*qnrS-R*	GTGAGTAATCGTATGTACTTTTGC<br>AAACACCTCGACTTAAGTCT	169	58
2	*intI1*	*intI-F*<br>*intI-R*	CGAACGAGTGGCGGAGGGTG<br>TACCCGAGAGCTTGGCACCCA	312	58
3	*tetM*	*tetM-F*<br>*tetM-R*	CATCATAGACACGCCAGGACATAT<br>CGCCATCTTTTGCAGAAATCA	101	58
4	*sul1*	*sul1-F*<br>*sul1-R*	CACCGGAAACATCGCTGCA<br>AAGTTCCGCCGCAAGGCT	158	58
5	*sul2*	*sul2-F*<br>*sul2-R*	GTCAAAGAACGCCGCAATGT<br>TCATCTGCCAAACTCGTCGTTA	105	58
6	*tetO*	*tetO-F*<br>*tetO-R*	CAACATTAACGGAAAGTTTATTGTATACCA<br>TTGACGCTCCAAATTCATTGTATC	104	60
7	*tetW*	*tetW-F*<br>*tetW-R*	ATGAACATTCCCACCGTTATCTTT<br>ATATCGGCGGAGAGCTTATCC	101	60
8	*ermA*	*ermA-F*<br>*ermA-R*	TTGAGAAGGGATTTGCGAAAAG<br>ATATCCATCTCCACCATTAATAGTAAACC	76	60
9	*ermB*	*ermB-F*<br>*ermB-R*	TAAAGGGCATTTAACGACGAAACT<br>TTTATACCTCTGTTTGTTAGGGAATTGAA	172	62
10	*qnrD*	*qnrD-F*<br>*qnrD-R*	GGAGCTGATTTTCGAGGG<br>AGAAAAATTAGCGTAACTAAGATTTGTC	105	62
11	16S rDNA	16S-F<br>16S-R	GGGTTGCGCTCGTTGC<br>ATGGYTGTCGTCAGCTCGTG	60	58

Table S7 Correlation coefficient statistics table of antibiotics and ARGs

	NOR	ENR	OFL	TC	CTC	DC	RTM	SD	SMX	SMD	*sul1*	*sul2*	*ermA*	*ermB*	*qnrD*	*qnrS*	*tetM*	*tetO*	*tetW*	intI1
NOR	1	.181	.231	.034	-.042	.344*	-.001	-.032	.422*	.526**	.174	.134	-.020	.233	-.075	.216	.129	.273	-.015	.125
ENR	.181	1	-.043	.009	-.005	-.214	.265	-.079	-.291	-.150	-.188	-.139	.412*	-.097	.265	-.147	-.083	-.106	.142	-.143
OFL	.231	-.043	1	.652**	.517**	.003	.330*	.083	.206	-.030	-.011	-.080	-.192	.056	-.177	.183	.033	-.015	-.107	-.022
TC	.034	.009	.652**	1	.962**	.097	.329	-.169	-.197	-.158	-.004	-.181	-.104	-.185	-.095	-.159	-.116	-.130	-.153	.041
CTC	-.042	-.005	.517**	.962**	1	.125	.362*	-.156	-.252	-.157	.050	-.166	-.095	-.170	-.087	-.147	-.107	-.120	-.141	.104
DC	.344*	-.214	.003	.097	.125	1	-.160	.243	.318	.370*	.460**	.274	.112	.568**	.121	.371*	.135	.259	.284	.528**
RTM	-.001	.265	.330*	.329	.362*	-.160	1	-.224	-.148	-.289	.037	-.035	-.085	.065	-.128	-.200	.221	-.280	-.134	-.062
SD	-.032	-.079	.083	-.169	-.156	.243	-.224	1	.309	-.238	.172	.250	.250	.253	.357*	.383*	.156	.386*	.527**	.288
SMX	.422*	-.291	.206	-.197	-.252	.318	-.148	.309	1	.653**	.352*	.295	-.062	.360*	.071	.427**	.272	.506**	.197	.378*
SMD	.526**	-.150	-.030	-.158	-.157	.370*	-.289	-.238	.653**	1	.258	.128	-.107	.244	-.156	.417*	.098	.353*	-.105	.291
sul1	.174	-.188	-.011	-.004	.050	.460**	.037	.172	.352*	.258	1	.586**	.136	.596**	.014	.374*	.443**	.520**	.185	.901**
sul2	.134	-.139	-.080	-.181	-.166	.274	-.035	.250	.295	.128	.586**	1	.162	.740**	.034	.439**	.901**	.262	.250	.646**
ermA	-.020	.412*	-.192	-.104	-.095	.112	-.085	.250	-.062	-.107	.136	.162	1	.235	.746**	.228	.084	.002	.765**	.051
ermB	.233	-.097	.056	-.185	-.170	.568**	.065	.253	.360*	.244	.596**	.740**	.235	1	.049	.626**	.702**	.138	.315	.520**
qnrD	-.075	.265	-.177	-.095	-.087	.121	-.128	.357*	.071	-.156	.014	.034	.746**	.049	1	.052	-.020	.125	.935**	-.004
qnrS	.216	-.147	.183	-.159	-.147	.371*	-.200	.383*	.427**	.417*	.374*	.439**	.228	.626**	.052	1	.399*	.185	.306	.446**
tetM	.129	-.083	.033	-.116	-.107	.135	.221	.156	.272	.098	.443**	.901**	.084	.702**	-.020	.399*	1	.125	.190	.489**
tetO	.273	-.106	-.015	-.130	-.120	.259	-.280	.386*	.506**	.353*	.520**	.262	.002	.138	.125	.185	.125	1	.200	.602**
tetW	-.015	.142	-.107	-.153	-.141	.284	-.134	.527**	.197	-.105	.185	.250	.765**	.315	.935**	.306	.190	.200	1	.148
intI1	.125	-.143	-.022	.041	.104	.528**	-.062	.288	.378*	.291	.901**	.646**	.051	.520**	-.004	.446**	.489**	.602**	.148	1

Note: *. It is marked in green at the level of 0.05; **. It is marked in red at the level of 0.01, and the correlation is significant.
